# Efficacy of single and repeated administration of ketamine in unipolar and bipolar depression: a meta-analysis of randomized clinical trials

**DOI:** 10.1007/s43440-020-00097-z

**Published:** 2020-04-16

**Authors:** Joanna Kryst, Paweł Kawalec, Alicja Mikrut Mitoraj, Andrzej Pilc, Władysław Lasoń, Tomasz Brzostek

**Affiliations:** 1 Laboratory of Pharmacology and Brain Biostructure, Maj Institute of Pharmacology, Polish Academy of Sciences, Kraków, Poland; 2 Department of Neurobiology, Maj Institute of Pharmacology, Polish Academy of Sciences, Kraków, Poland; 3 Departament of Nutrition and Drug Research, Faculty of Health Sciences, Institute of Public Health, Jagiellonian University, Kraków, Poland; 4 Kraków, Poland; 5 Department of Experimental Neuroendocrinology, Maj Institute of Pharmacology, Polish Academy of Sciences, Kraków, Poland; 6 Department of Internal and Community Nursing, Institute of Nursing and Midwifery, Faculty of Health Sciences, Jagiellonian University Medical College, Kraków, Poland

**Keywords:** Ketamine, Antidepressants, Major depressive disorder, Bipolar disorder, Meta-analysis, RCT

## Abstract

**Background:**

Due to unmet clinical needs for efficient drugs with a rapid onset of antidepressant effects, we aimed to evaluate the efficacy of single-dose ketamine in different subgroups of patients with major depression and establish whether repeated ketamine administration could be a viable strategy to maintain treatment gains.

**Methods:**

Electronic databases (Medline via PubMed, Embase, Cochrane Library, Trip Database) were systematically searched until February 22, 2019, for published peer-reviewed randomized controlled trials (RCTs) concerning a single and repeated administration of ketamine in patients with major depression. All relevant RCTs were selected and critically appraised, and a meta-analysis of eligible studies was performed.

**Results:**

A total of 20 studies were included in the meta-analysis. The largest effect of ketamine vs. controls in reducing depressive symptoms was observed at 24 h (SMD = − 0.89; 95% CI − 1.24; − 0.53; *p* < 0.00001); however, a significant difference was shown for up to 7 days after a single dose. Significant differences compared with controls were observed for up to 7 days in treatment-resistant patients and when ketamine was added to ongoing antidepressant treatment, while there were no significant differences at 7 days when ketamine was used as monotherapy. In patients with major depression, initial antidepressant effects of ketamine were maintained during repeated dosing. At 2–3 weeks of repeated ketamine treatment, significant reduction of depression severity scores was observed: SMD = − 0.70; 95% CI − 1.15; − 0.25 or SMD = − 0.81; 95% CI − 1.41; − 0.20 (depending on the dosing regimen used); *p* ≤ 0.009 vs placebo.

**Conclusions:**

Our meta-analysis revealed rapid and robust antidepressant effects of single-dose ketamine in patients with treatment-resistant depression (TRD). By pooling data from RCTs, we showed for the first time that repeated ketamine administration is effective in sustaining initial antidepressant effects observed after single dosing.

**Electronic supplementary material:**

The online version of this article (10.1007/s43440-020-00097-z) contains supplementary material, which is available to authorized users.

## Introduction

Depression is a common mental disorder and a leading cause of disability worldwide [1]. According to World Health Organization data, more than 264 million people globally are affected by depression, and the disease burden has been increasing worldwide [2]. There are a number of well-established treatments for major depressive disorder (MDD), but they are only partially effective or even not effective at all in a significant number of patients [3]. Despite multiple treatment approaches, about 30% of patients with MDD do not respond to conventional antidepressants and remain symptomatic [3]. Another limitation of the existing antidepressants is a delayed onset of action [4], which can result in treatment cessation and increased suicidal risk in some patients [3, 5]. As a consequence, there is a critical need for new rapidly acting and effective treatment options for people with both unipolar and bipolar depression.

Ketamine is a noncompetitive *N*-methyl-d-aspartate glutamate receptor antagonist originally approved for use as an anesthetic. In 2000, Berman et al. [6], for the first time showed rapid and robust antidepressant effects of single-dose ketamine, confirmed later in numerous clinical studies among patients with major depression [7–9]. Importantly, ketamine is effective also in treatment-resistant depression (TRD), which is mostly defined as lack of response to at least two different antidepressant drugs at an adequate dose and duration of administration [10]. However, antidepressant effects of single ketamine administration are short lived, and the average duration of response after a single dose is around 1 week [11]. Data from open-label studies showed promising results of repeated ketamine administration [12, 13] and a trend to a more pronounced reduction in depression severity after successive ketamine infusions [14, 15]. In 2018, the first results of research on repeated intranasal administration of esketamine (the *S*-enantiomer of ketamine) has shown a significant improvement of depressive symptoms in patients with TRD, which has been sustained for up to 9 weeks of treatment [16].

Ketamine effects in depression have been studied in previous meta-analyses [11, 17–19]. However, in the last few years, new randomized controlled trials (RCTs) regarding ketamine use in major depression have been conducted, some of which assessed repeated ketamine administration. Due to a growing body of evidence from RCTs, there is a need for a meta-analysis that would elucidate the antidepressant effects of ketamine in selected groups of patients (e.g., TRD, patients treated with ketamine alone or in combination with ongoing antidepressant treatment) as well as establish whether repeated ketamine administration could be a viable strategy to maintain treatment gains.

## Methods

### Literature search strategy

A systematic review was performed using the following databases: Embase, Medline (via PubMed), Cochrane Central, and Trip Database until February 22, 2019. The search strategy was based on the MeSH (medical subject heading) terms and Emtree, combined with Boole’s logical operators with major search terms “ketamine” AND “depression” (Table 1) and supplemented with hand-searching reference lists of identified studies. Clinical trials registries (www.clinicaltrials.gov, www.clinicaltrialsregister.eu), identified systematic reviews, and meta-analyses were also searched for relevant data.

**Table 1:** MeSH subject headings and EMTREE keywords used in search strategy construction (last updated: 22.02.2019)

	Keywords (combined with boolean logical operators: AND, OR)
Medical condition	(Depressive Disorders) OR (Depressive Disorder) OR (Depressive Neurosis) OR (Depressive Neuroses) OR (Endogenous Depression) OR (Endogenous Depressions) OR (Depressive Syndrome) OR (Depressive Syndromes) OR Depression OR (Neurotic Depression) OR (Neurotic Depressions) OR Melancholia OR Melancholias OR (Unipolar Depression) OR (Unipolar Depressions) OR Bipolar Disorder OR MDD OR (Major Depressive Disorders) OR (Major Depressive Disorder)
Intervention	(Ketamine Hydrochloride) OR Ketamine OR Calipsol OR Kalipsol OR Calypsol OR Ketalar OR Ketaset OR Ketanest OR CI-581 OR CI 581 OR CI581
Methodological limits	PubMed: Humans, Controlled Clinical Trial, Randomized Controlled Trial EMBASE: Humans, Controlled Clinical Trial, Randomized Controlled Trial CENTRAL: Cochrane Central Register of Controlled Trials, Word variations have been searched

### Selection criteria

Two independent contributors (J.K. and A.M.M.) used the same search strategy to identity relevant clinical trials. All disagreements were resolved by discussion with the third author (P.K.) to reach consensus. The study selection was based on the titles and abstracts, and, finally, on full-text articles. The meta-analysis included RCTs comparing ketamine with placebo or “active placebo” (non-antidepressant anesthetic) in patients with major depression. All relevant RCTs were selected and critically appraised according to the Preferred Reporting Items for Systematic Reviews and Meta-Analyses (PRISMA) statement [20]. The following criteria were used for study inclusion: (1) RCTs (crossover or parallel) assessing more than 5 patients; (2) adult patients treated for major depression (unipolar or bipolar, treatment-resistant or not), with no restrictions on concomitant pharmacological or psychological treatments; (3) placebo or non-antidepressant anesthetic as a comparator; (4) ketamine therapy at a fixed dose (single or repeated administration—if repeated administration was studied results reported at least 2 weeks after the start of repeated dosing), with no restriction on the ketamine regimen used (e.g., dose or route); (5) evaluation of depression severity based on a validated scale; (6) English-language papers. Studies were excluded based on the following criteria: (1) trials conducted in the context of electroconvulsive therapy (ECT) and surgery; (2) patients with “narrow” (e.g., postpartum depression) or secondary depression diagnoses (e.g., vascular depression); (3) ketamine used in an ascending dose; (4) duplicate studies. Data reported only in abstract form (with no associated full text) were rejected due to the lack of detailed information about methodology, population, and results. Nonrandomized as well as uncontrolled open-label studies and case reports were not included.

### Data extraction and outcome measure

Data extracted by the first author (J.K.) were verified by the second author (A.M.M.). The primary outcome was a change from baseline in depression severity scores on depression scales such as the Hamilton Depression Rating Scale (HDRS) and/or the Montgomery–Åsberg Depression Rating Scale (MADRS) at the following time points: (1) day 1 (24 h); day 3 ([21] reported data for day 4); day 7 after a single administration; (2) 2–4 weeks after the start of repeated dosing. The above time points were also used to assess response to treatment and remission. The following data were also extracted: population characteristics, study design, details of intervention and regimen, and definition of outcomes. If different doses of intravenous (IV) ketamine were analyzed in the study, the results for the dose of 0.5 mg/kg were considered, as a recent meta-analysis has shown a lower antidepressant effect of very low doses of ketamine (0.1–0.4 mg/kg IV) [22]. For trials with a crossover design, results from the first period prior to the crossover were searched in published Cochrane meta-analyses [17, 18] or were requested from the authors of primary studies. Missing data were calculated from graphs independently by two authors and searched in clinical trials registry (www.clinicaltrials.gov).

### Data analysis

Potential sources of bias were identified for each trial, using the criteria recommended in the Cochrane Handbook [23]. For continuous outcomes, the standardized mean difference (SMD) between ketamine and comparator with 95% confidence intervals (CIs) was calculated. SMD was used as the included studies measured depressive symptoms in different psychometric scales. For dichotomous outcomes, the odds ratio (OR) with 95% CIs was calculated. The random effects model for both dichotomous and continuous variables was applied, because it has the highest generalizability for empirical examination of summary effect measures in meta-analyses [24]. Statistical significance was defined at a *p* value of less than 0.05. The results were presented as forest plots, using Review Manager v.5.3 and Microsoft Excel®. Sensitivity analysis was scheduled for the primary outcome as leave-one-study-out and exclusion of crossover trials if data regarding the first period before the crossover were not obtained. Subgroup analysis was planned for TRD, ketamine as monotherapy and as add-on to ongoing antidepressant therapy, placebo- and midazolam-controlled trials, unipolar and bipolar depression.

## Results

### Search results

**Fig. 1:**
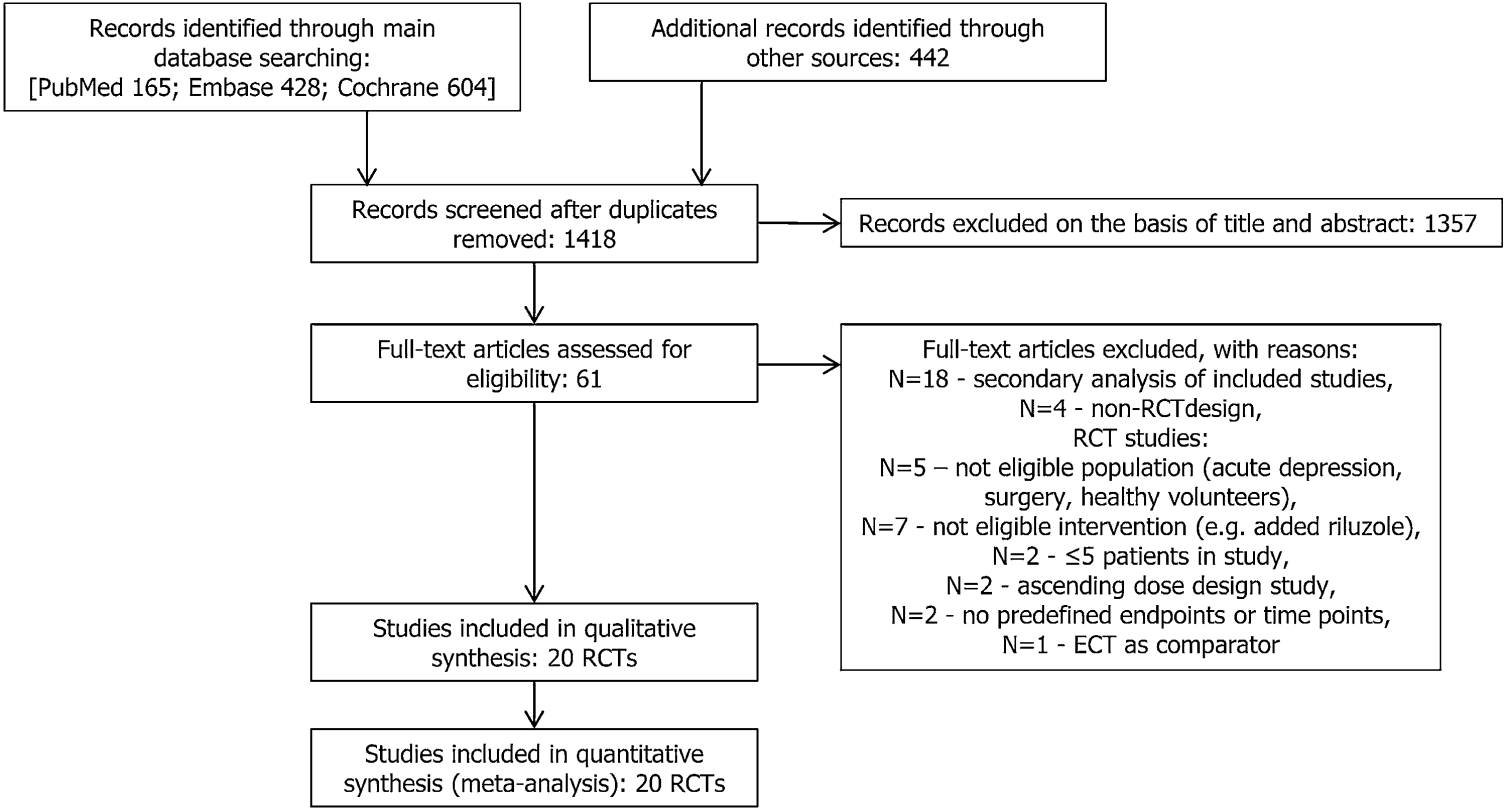
PRISMA flow diagram for selection of studies identified in the systematic review

**Fig. 2:**
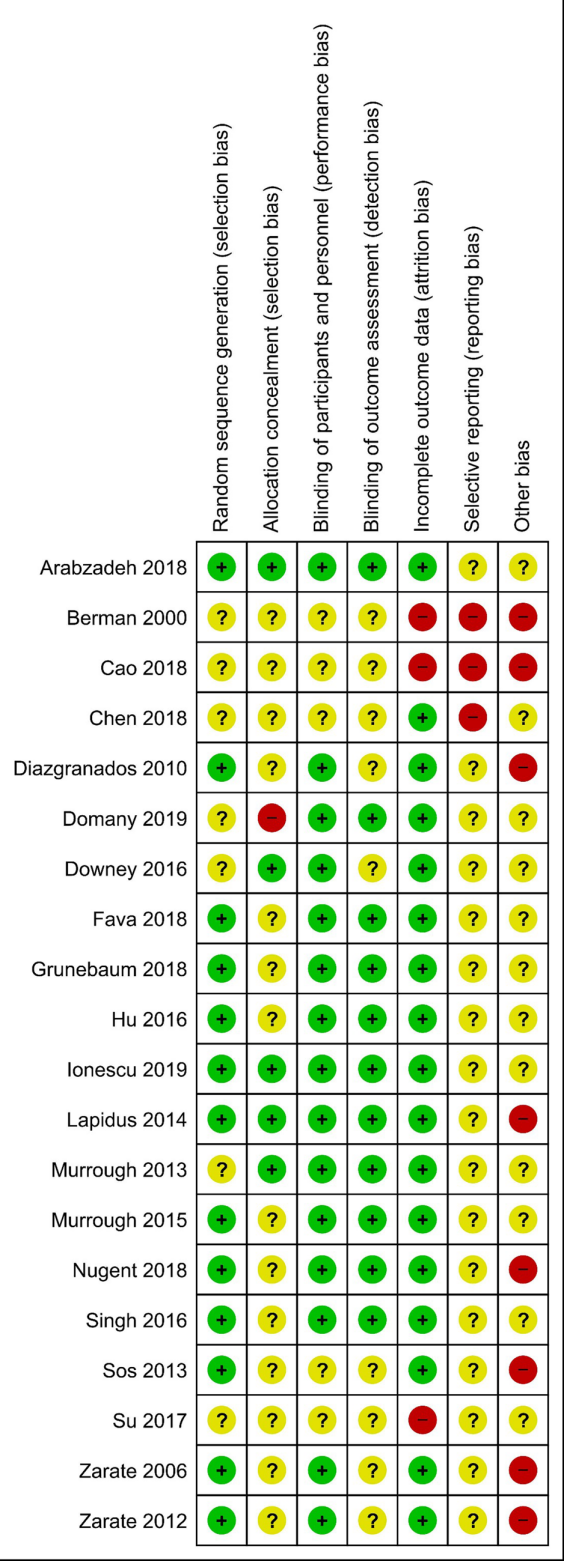
Risk of bias summary: review authors’ judgements about each risk of bias item for each included study

The electronic searches yielded 1418 items after duplicates were removed. The selection of titles and abstracts resulted in 61 potentially relevant articles, of which 41 were excluded due to the reasons presented in Fig. 1. Finally, 20 studies described in 21 references met the predefined inclusion criteria. Of the 20 included studies, 16 trials investigating the clinical effects of a single-dose ketamine and 4 assessing the effects of repeated administration were suitable for quantitative synthesis (meta-analysis). The flow of information through the different phases of the systematic review is shown in Fig. 1. The methodology and main characteristics of patients involved in each RCT are described in Table 2. Potential sources of bias are summarized in Fig. 2.

**Table 2:** Characteristics of included randomized clinical trials of ketamine

Study	Methodology	Sample	Age	Intervention	Control	Dosing	Population	Concomitant therapy	Scale/baseline scale score
Single administration									
Berman et al. 2000 [6]	RCT, DB, crossover (1-week washout), United States	9	23–56	IV ketamine 0.5 mg/kg	IV placebo	Single infusion (40 min)	MDD (11.1% with BD), TRD not reported	Monotherapy	HDRS-25/NA
Zarate et al. 2006 [7]	RCT, DB, crossover (1-week washout), 1 site in the United States	18	18–65	IV ketamine 0.5 mg/kg	IV placebo	Single infusion (40 min)	MDD (BD excluded), TRD*	Monotherapy	HDRS-21/ ≥ 18
Diazgranados et al. 2010 [8]	RCT, DB, crossover (2-week washout), 1 site in the United States	18	18–65	IV ketamine 0.5 mg/kg	IV placebo	Single infusion (40 min)	BD, TRD**	Add-on to therapeutic levels of lithium or valproate (no other psychotropic medications allowed)	MADRS/ ≥ 20
Zarate et al. 2012 [9]	RCT, DB, crossover (2-week washout), 1 site in the United States	15	18–65	IV ketamine 0.5 mg/kg	IV placebo	Single infusion (40 min)	BD, TRD**	Add-on to therapeutic levels of lithium or valproate (no other psychotropic medications allowed)	MADRS/ ≥ 20
Murrough et al. 2013 [68]	RCT, DB, parallel, 2 sites in the United States	73	21–80	IV ketamine 0.5 mg/kg	IV midazolam 0.045 mg/kg	Single infusion (40 min)	MDD (BD excluded), TRD*	Monotherapy (nonbenzodiazepine hypnotic allowed)	MADRS/NA
Sos et al. 2013 [21]	RCT, DB, crossover (1-week washout), 1 site in Czech Republic	30	18–65	IV ketamine 0.54 mg/kg	IV placebo	Single infusion; loading dose of 0.27 mg/kg for the first 10 min, then the same dose within 20 min	MDD, TRD not reported	Add-on to a stable dose of AD for ≥ 3 weeks	MADRS/ ≥ 20
Lapidus et al. 2014 [25]	RCT, DB, crossover, (1-week washout), 1 site in the United States	20	21–65	intranasal ketamine 50 mg	intranasal placebo	Single (5 administrations of 10 mg over 20 min)	MDD (BP excluded), TRD***	Add-on to a stable dose of AD	MADRS / NA
Murrough et al. 2015 [51]	RCT, DB, parallel, 1 site in the United States	24	18–80	IV ketamine 0.5 mg/kg	IV midazolam 0.045 mg/kg	Single infusion (40 min)	Mood and anxiety spectrum disorders: MDD (54%), BD (29%), patients with suicidal ideation, TRD not reported	Add-on to a stable dose of psychotropic medication including AD	MADRS / NA
Downey et al. 2016 [27]	RCT, DB, parallel, 2 sites in the United Kingdom	60	18–45	IV ketamine 0.5 mg/kg, lanicemine 100 mg	IV placebo	Single infusion (60 min)	MDD (BP excluded), TRD not reported	Monotherapy	MADRS/ ≥ 20
Hu et al. 2016 [69]	RCT, DB, parallel, 1 site in China	30	18–60	IV ketamine 0.5 mg/kg	IV placebo	Single infusion (40 min)	MDD (BD excluded), 55.6% TRD*	Add-on to newly initiated 4-week therapy with escitalopram (10 mg/day), additionally only zolpidem allowed	HDRS-17/ ≥ 24
Su et al. 2017 [70], Chen et al. 2018 [71]	RCT, DB, parallel, 1 site in Taiwan	71	21–65	IV ketamine (0.2; 0.5 mg/kg)	IV placebo	Single infusion (40 min)	MDD (BP excluded), TRD*	Add-on to AD	HDRS-17/ ≥ 18
Cao et al. 2018 [28]	RCT, DB, parallel, 1 site in Taiwan	55	NA, mean baseline ≥ 46 years	IV ketamine (0.2; 0.5 mg/kg)	IV placebo	Single infusion (40 min)	MDD (BP excluded), TRD*	Add-on to AD	HDRS-17 / ≥ 18
Chen et al. 2018 [72]	RCT, DB, parallel, Taiwan	24	21–65	IV ketamine (0.2; 0.5 mg/kg)	IV placebo	Single infusion (40 min)	MDD, TRD*	Add-on to a stable AD for ≥ 2 weeks	HDRS-17 / NA
Fava et al. 2018 [67]	RCT, DB, parallel, 6 sites in the United States	99	18–70	IV ketamine (0.1; 0.2; 0.5 and 1.0 mg/kg)	IV midazolam 0.045 mg/kg	Single infusion (40 min)	MDD (BP excluded), TRD*	Add-on to a stable AD for ≥ 4 weeks	MADRS / ≥ 20
Grunebaum et al. 2018 [29]	RCT, DB, parallel, 1 site in the United States	80	18–65	IV ketamine 0.5 mg/kg	IV midazolam 0.02 mg/kg	Single infusion (40 min)	MDD, patients with suicidal ideation, TRD not reported	Add-on to AD (benzodiazepines not allowed within 24 h before the infusion)	HDRS-17/ ≥ 16
Nugent et al. 2018 [26]	RCT, DB, crossover, 1 site in the United States	35	18–65	IV ketamine 0.5 mg/kg	IV placebo	Single infusion (40 min)	MDD, (BP excluded), TRD***	Monotherapy	MADRS/ ≥ 20
Repeated administration									
Singh et al. 2016 [32]	RCT, DB, parallel, 14 sites in the United States	68	18–64	IV ketamine 0.5 mg/kg	IV placebo	2 or 3 infusions (40 min) per week for 4 weeks	MDD (BD excluded), TRD*	Add-on to a stable dose of AD	MADRS/NA
Arabzadeh et al. 2018 [33]	RCT, DB, parallel, 1 site in Iran	90	18–60	Oral ketamine 50 mg/day	Oral placebo	2 times a day (25 mg) for 6 weeks	MDD, TRD not reported	Added to newly initiated therapy of sertraline (150 mg/day)	HDRS / ≥ 20
Domany et al. 2019 [31]	RCT, DB, parallel, 1 site in Israel	41	18–75	Oral ketamine 1 mg/kg	Oral placebo	3 times per week for 3 weeks	MDD (BP excluded), TRD*	Add-on to AD	MADRS/ ≥ 19
Ionescu et al. 2019 [30]	RCT, DB, parallel, 1 site in the United States	26	18–65	IV ketamine 0.5 mg/kg	IV placebo	2 infusions (45 min) per week for 3 weeks	MDD (BP excluded), patients with suicidal ideation, TRD*	Add-on to a stable AD for ≥ 4 weeks	HDRS-28/ ≥ 20

*AD* antidepressants, *BD* bipolar disorder, *DB* double blinded, *HDRS* Hamilton Depression Rating Scale, *IV* intravenous, *MADRS* Montgomery–Åsberg Depression Rating Scale, *MDD* major depressive disorder, *NA* not available, *RCT *randomized controlled trial, *TRD *treatment-resistant depression

*Failed at least 2 adequate antidepressant trials

**Failed at least 1 adequate antidepressant trial and failed to respond to a prospective open trial of therapeutic levels of either lithium or valproate

***Failed at least 1 adequate antidepressant trial

### Single doses: effects on depression severity scores over time

**Fig. 3:**
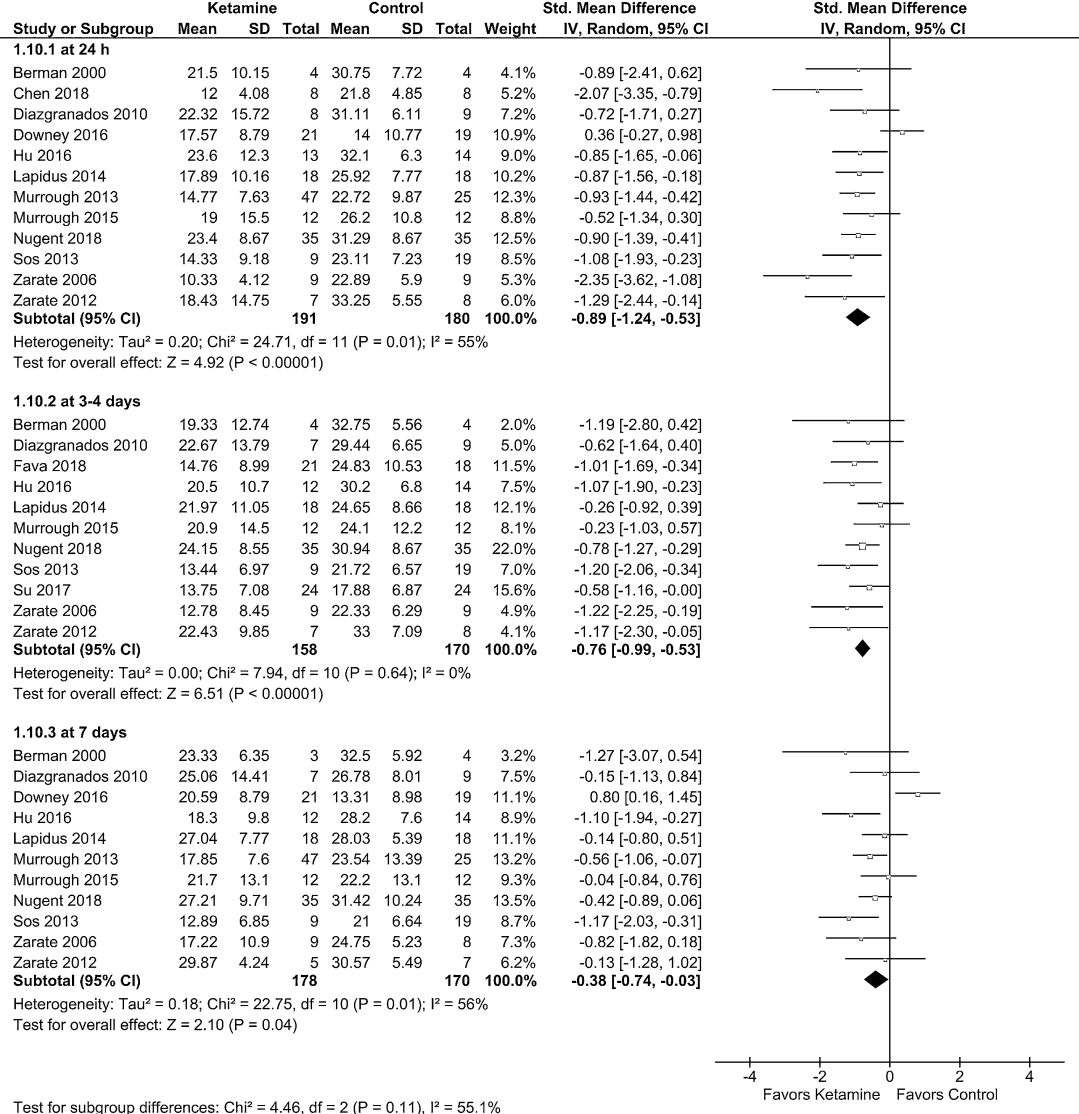
Effects of single-dose ketamine on depression rating scale at 24 h, 3–4 days, and 7 days

Based on pooled data from the included studies, single-dose ketamine results in a significant reduction of depression severity scores at 24 h (SMD = − 0.89 [95% CI − 1.24; − 0.53]; *p* < 0.00001; test for heterogeneity: Chi^2^ = 24.71; *df* = 11; *p* = 0.01; I^2^ = 55%), 3–4 days (SMD =− 0.76 [95% CI − 0.99; − 0.53]; *p* < 0.00001; test for heterogeneity: Chi^2^ = 7.94; *df* = 10; *p* = 0.64; *I*^2^ = 0%), and 7 days (SMD = − 0.38 [95% CI − 0.74; − 0.03]; *p* = 0.04; test for heterogeneity: Chi^2^ = 22.75; *df* = 10; *p* = 0.01; *I*^2^ = 56%) in comparison with placebo, with the largest effect at 24 h postadministration (Fig. 3). The sensitivity analysis after the exclusion of studies for which data relating to the period before the crossover were not obtained [25, 26] showed that the observed effect was not markedly affected; however, the statistical difference was lost for 7 days. The forest plot for all time points and results of the sensitivity analysis are presented in Fig. 3 and Table 3. Leave-one-out analyses showed a more pronounced reduction of depression severity scores at 24 h (SMD = − 0.98 [95% CI − 1.21; − 0.74]; *p* < 0.00001; test for heterogeneity: Chi^2^ = 9.43; *df* = 10; *p* = 0.49; *I*^2^ = 0%) and 7 days (SMD = − 0.50 [95% CI − 0.74; − 0.27]; *p* < 0.0001; test for heterogeneity: Chi^2^ = 8.95; *df *= 9; *p* = 0.44; *I*^2^ = 0%) after exclusion of the study by Downey et al. [27], which showed no antidepressant effects of ketamine vs. control. Ketamine administration was shown to improve mostly emotional symptoms. Its effects on the individual HDRS/MDRS symptoms reported in the primary RCTs are presented in Table 4.

**Table 3:** Sensitivity and subgroup analysis—effects of single-dose ketamine on depression rating scale at 24 h, 3–4 days, and 7 days

Time point/subgroup^^^	Standardized mean difference (SMD)							
	Ketamine monotherapy*	Ketamine add-on to AD*	TRD*	Bipolar depression*	Unipolar depression*	Midazolam-controlled trials*	Placebo-controlled trials*	Sensitivity analysis*^,^^^
24 h	− 0.83 [− 1.55, − 0.12]; *p* = 0.02; test for heterogeneity: Chi^2^ = 18.94, *df* = 4 (*p* = 0.0008); *I*^2^ = 79%	− 0.94 [− 1.27, − 0.61]; *p* < 0.00001; test for heterogeneity: Chi^2^ = 4.73, *df* = 6 (*p* = 0.58); *I*^2^ = 0%	− 1.19 [− 1.65, − 0.73]; *p* < 0.00001; test for heterogeneity: Chi^2^ = 7.40, *df* = 5 (*p* = 0.19); *I*^2^ = 32%	− 0.96 [− 1.71, − 0.21]; *p* = 0.01; test for heterogeneity: Chi^2^ = 0.55, df = 1 (*p* = 0.46); *I*^2^ = 0%	− 0.89 [− 1.29, − 0.48]; *p* < 0.0001; test for heterogeneity: Chi^2^ = 23.99, *df* = 9 (*p* = 0.004); *I*^2^ = 62%	− 0.81 [− 1.25, − 0.38]; *p* = 0.0002; Chi^2^ = 0.69, *df* = 1 (*p* = 0.41); *I*^2^ = 0%	− 0.95 [− 1.40, − 0.50]; *p* < 0.0001; test for heterogeneity: Chi^2^ = 24.01, *df* = 9 (*p* = 0.004); *I*^2^ = 63%	− 0.92 [− 1.39,− 0.46]; *p* = 0.0001; Chi^2^ = 24.49, *df* = 9 (*p* = 0.004); *I*^2^ = 63%
3−4 days	− 0.88 [− 1.31, − 0.46]; *p* < 0.0001; test for heterogeneity: Chi^2^ = 0.72, *df* = 2 (*p* = 0.70); *I*^2^ = 0%	− 0.71 [− 0.98, − 0.44]; *p* < 0.00001; test for heterogeneity: Chi^2^ = 6.74, *df* = 7 (*p* = 0.46); *I*^2^ = 0%	− 0.88 [− 1.21, − 0.55]; *p* < 0.00001; test for heterogeneity: Chi^2^ = 2.30, df = 5 (*p* = 0.81); I^2^ = 0%	− 0.87 [− 1.62, − 0.11]; *p* = 0.02; test for heterogeneity: Chi^2^ = 0.51, *df* = 1 (*p* = 0.47); *I*^2^ = 0%	− 0.75 [− 0.99, − 0.51]; *p* < 0.00001; test for heterogeneity: Chi^2^ = 7.33, *df* = 8 (*p* = 0.50); *I*^2^ = 0%	− 0.65 [− 1.42, 0.11]; *p* = 0.09; test for heterogeneity: Chi^2^ = 2.15, *df* = 1 (*p* = 0.14);* I*^2^ = 53%	− 0.77 [− 1.03, − 0.52]; *p* < 0.00001; test for heterogeneity: Chi^2^ = 5.71, *df* = 8 (*p* = 0.68);* I*^2^ = 0%	− 0.84 [− 1.12, − 0.56]; *p* < 0.00001; test for heterogeneity: Chi^2^ = 5.41, *df* = 8 (*p* = 0.71); *I*^2^ = 0%
7 days	− 0.31 [− 0.93, 0.30]; *p* = 0.32; test for heterogeneity: Chi^2^ = 14.61, *df* = 4 (*p* = 0.006); *I*^2^ = 73%	− 0.46 [− 0.89, − 0.03]; *p* = 0.04; test for heterogeneity: Chi^2^ = 7.60, *df* = 5 (*p* = 0.18); *I*^2^ = 34%	− 0.60 [v0.94, − 0.25]; *p* = 0.0008; test for heterogeneity: Chi^2^ = 3.06, *df* = 4 (*p* = 0.55); *I*^2^ = 0%	− 0.14 [− 0.89, 0.61]; *p* = 0.72; test for heterogeneity: Chi^2^ = 0.00, *df* = 1 (*p* = 0.98); I^2^ = 0%	− 0.43 [− 0.84, − 0.02]; *p* = 0.04; test for heterogeneity: Chi^2^ = 22.41, *df* = 8 (*p* = 0.004); *I*^2^ = 64%	− 0.40 [− 0.88, 0.08]; *p* = 0.10; test for heterogeneity: Chi^2^ = 1.21, *df* = 1 (*p* = 0.27); *I*^2^ = 17%	− 0.41 [− 0.86, 0.05]; *p* = 0.08; test for heterogeneity: Chi^2^ = 21.40, *df* = 8 (*p* = 0.006); *I*^2^ = 63%	− 0.42 [− 0.90, 0.05]; *p* = 0.08; test for heterogeneity: Chi^2^ = 22.28, *df* = 8 (*p* = 0.004); *I*^2^ = 64%

*No significant difference (*p* > 0.05) between ketamine vs control at baseline. *AD* antidepressant therapy, *TRD* treatment-resistant depression

^^^Subgroup analysis: ketamine monotherapy: [6, 7, 26, 27, 68]; ketamine add-on to AD—all studies excluding: [6, 7, 26, 27, 68]; TRD—lack of response to at least 2 different antidepressant drugs at an adequate dose and duration of administration: [7–9, 28, 67–70, 72]; bipolar depression: [8, 9]; unipolar depression: all studies excluding: [8, 9] (although [6] and [51] included some patients with bipolar depression most subjects in these studies suffered from unipolar depression, respectively, 88.9% and 54%); midazolam-controlled trials: [29, 51, 67, 68]; placebo-controlled trials: all studies excluding: [29, 51, 67, 68]

^^^^Sensitivity analysis: crossover studies for which data regarding the first period before crossover were not obtained: [25, 26]

**Table 4:** Ketamine effects on the individual HDRS/MADRS symptoms in primary RCTs

Study	Scale	Ketamine-treated patients
Berman et al. 2000 [6]	HDRS-25	Significant improvement for items of depressed mood, suicidality, helplessness, worthlessness
Zarate et al. 2006 [7]	HDRS-21	Significant improvement (time–drug interaction) for items of depressed mood, guilt, work and interests and psychic anxiety significant improvement (main effect for drug) for items of suicide, insomnia, general somatic symptoms, genital symptoms, and hypochondriasis deterioration of depersonalization or derealization from 40 to 110 min and motor retardation and gastrointestinal symptoms at 40 min (improvement in motor retardation at day 1)
Diazgranados et al. 2010 [8]	MADRS	Significant improvement for items of apparent sadness, reported sadness, inner tension, reduced appetite, concentration difficulties, lassitude, inability to feel, pessimistic thoughts
Zarate et al. 2012 [9]	MADRS	Significant improvement for items of apparent sadness, reported sadness, inner tension, concentration difficulties, lassitude, inability to feel, pessimistic thoughts, suicidal thoughts
Su et al. 2017 [70]	HDRS-17	Significant differences observed for total HDRS score was due to changes on emotional symptoms (somatic anxiety, psychological anxiety, guilt and delusions, loss of interest, depressed mood) persisting throughout the study and rapid but short-lasting changes on atypical symptoms (reduced libido, psychomotor slowing, suicidality, psychomotor agitation, hypochondriasis) but not insomnia-related symptoms (energy/fatigability, delayed sleep onset, midnocturnal awakening, early morning awakening)

### Single doses: effects on response and remission rates over time

**Fig. 4:**
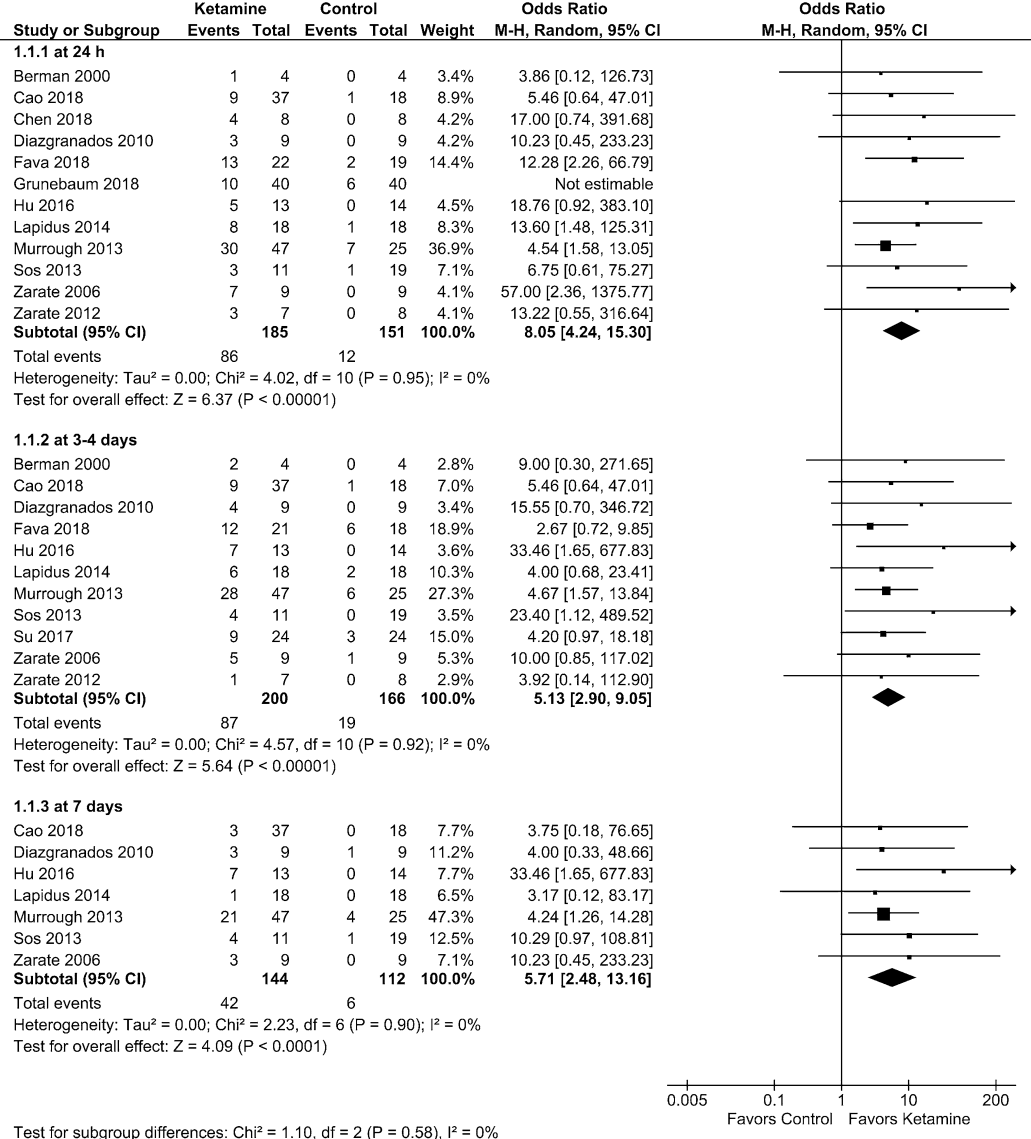
Effects of single-dose ketamine on response rates at 24 h (sensitivity analysis after exclusion of the study by Grunebaum et al. [29]), 3–4 days, and 7 days

In included RCTs, response was mostly defined as ≥ 50% reduction in Depression Severity Score from baseline (≥ 45% in [28]), while remission, as MADRS Score ≤ 10. Pooled results of 12 RCTs providing data on the 24-h response rate showed a low treatment effect on response (OR = 5.64 [95% CI 3.23; 9.85]; *p* < 0.00001; test for heterogeneity: Chi^2^ = 8.89, *df *= 11; *p* = 0.63; *I*^2^ = 0%). Moreover, the effect of treatment on response was not consistent with the pooled data of a reduction in depression severity scores over time. While continuous outcomes showed the largest effect at 24 h postadministration, pooled ORs for response at 24 h was similar to those at 3–4 days (OR = 5.13 [95% CI 2.90; 9.05]; *p* < 0.00001) and 7 days (OR = 5.71 [95% CI 2.48; 13.16]; *p* < 0.0001) (Fig. 4). Leave-one-out analyses showed that after exclusion of a study involving patients with suicidal thoughts [29], the pooled OR for response at 24 h (OR = 8.05 [95% CI 4.24; 15.30]; *p* < 0.00001) (Fig. 4) corresponded with results of the meta-analysis showing the most pronounced reduction in depressive symptoms (vs. controls) at 24 h postadministration. Of note, the response rate at 24 h postadministration was the only data reported by Grunebaum et al. [29] that were useful for meta-analysis, and the meta-analysis of any other outcomes did not confound with the results of this trial.

**Fig. 5:**
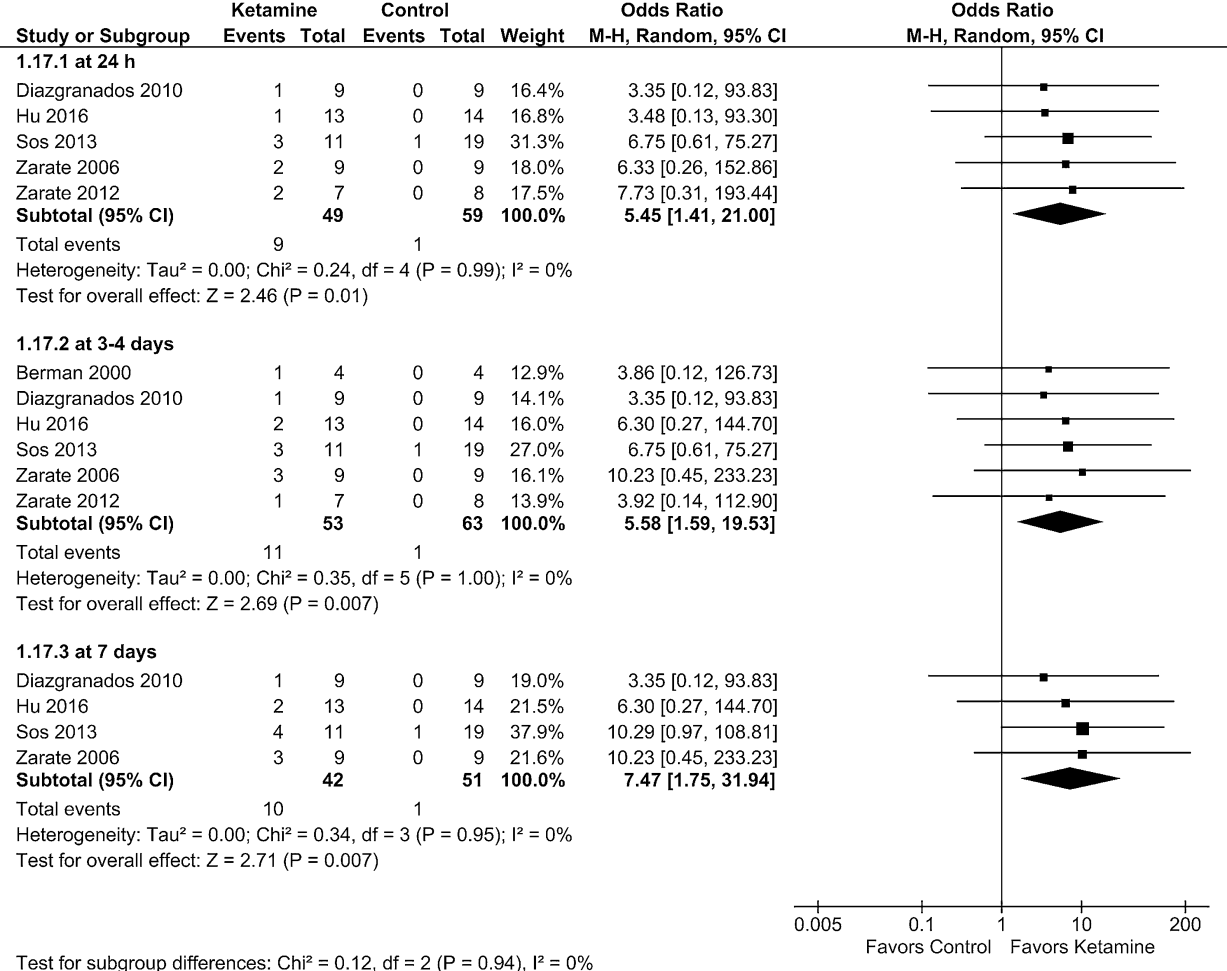
Effects of single-dose ketamine on remission rates at 24 h, 3–4 days, and 7 days

Pooled data showed a significant difference in remission in favor of ketamine over the control group at 24 h, 3–4 days, and 7 days postadministration (Fig. 5). Although the pooled ORs for remission at day 7 were higher than those reported at 24 h and 3–4 days, sensitivity analyses revealed no marked difference after exclusion of each single trial, showing that the overall results were not driven by one study. As the remission rate remained consistent throughout the time points, it can be suggested that ketamine efficacy could be more stable and longer in early remitters than in early responders.

### Single doses: a subgroup analysis

**Fig. 6:**
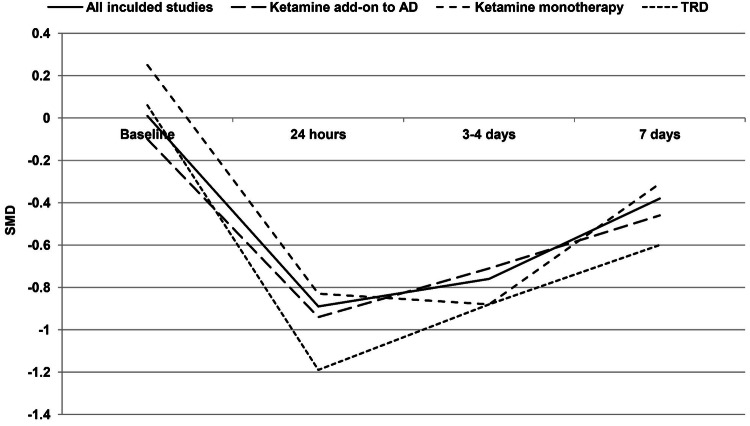
Meta-analysis results: time course of overall standardized mean differences (SMD) between ketamine and control in major depression—a subgroup analysis (abbreviations, see Table 2)

Subgroup analyses showed significant advantages of ketamine over controls in reducing depressive symptoms from 24 h to 7 days postadministration in treatment-resistant patients and when ketamine was used as add-on to ongoing antidepressant treatment and from 24 h to 3–4 days when ketamine was used as monotherapy (Table 3). The most pronounced effect favoring ketamine over controls was observed in patients with TRD (Fig. 6). Ketamine produced also a significant reduction of depression severity scores from 24 h to 7 days (vs. controls) in unipolar depression. In bipolar depression, between-group differences were significant at 24 h and 3 days postadministration; however, only two RCTs assessing the antidepressant effects of ketamine in patients exclusively with bipolar depression were included [8, 9]. Detailed data of all subgroup analyses are reported in Table 3.

Subgroup analyses were also performed to assess the influence of the control arm (placebo or active control—midazolam) on the antidepressant effect size of ketamine. In four studies, midazolam was used in a control group; however, the results of individual meta-analyses were based on no more than two studies owing to limited available data for the time points analyzed. The advantages of ketamine over control in reducing depressive symptoms were shown both at 24 h and 3–4 days in placebo-controlled trials and only at 24 h postadministration in midazolam-controlled trials, with no significant difference at 7 days, regardless of control group (Table 3). Similarly, in midazolam-controlled trials, the pooled OR for response at 24 h was less pronounced (OR = 4.09 [95% CI 1.58; 10.54]) when compared with pooled data from all included RCTs (see Fig. 4), even after exclusion of the results of Grunebaum et al. [29] (OR = 6.00 [95% CI 2.45; 14.70]) due to the reasons described earlier. The above data are consistent with higher response rates at 24 h in midazolam-control arms (11–28%) than placebo-control arms (0–6%), reported in primary studies included in the meta-analysis.

### Repeated doses

Among the four identified RCTs of repeated ketamine administration, we searched for the results reported for the last observation during the randomized phase of each study. That was 3 weeks in the studies by Ionescu et al. [30] and Domany et al. [31], and 4 weeks in the study by Singh et al. [32]. However, Singh et al. [32] reported that most patients from placebo groups crossed to ketamine treatment after day 15 according to the predefined protocol (due to lack of efficacy). Therefore, data reported at day 15 (2 weeks) of the randomized phase were included in the meta-analysis. Arabzadeh et al. [33] reported efficacy results for as long as 6 weeks; however, as patients in this study newly initiated sertraline therapy, only 2-week data would not be biased by the onset of sertraline antidepressant effects [34]. As a result, data after 2–3 weeks of repeated ketamine administration from particular studies were available for the meta-analysis. Singh et al. [32] reported that the twice- and thrice-weekly ketamine dosing regimens used in the study were equally effective in sustaining the antidepressant response; however, a separate meta-analysis was performed for both treatment regimens.

**Fig. 7:**
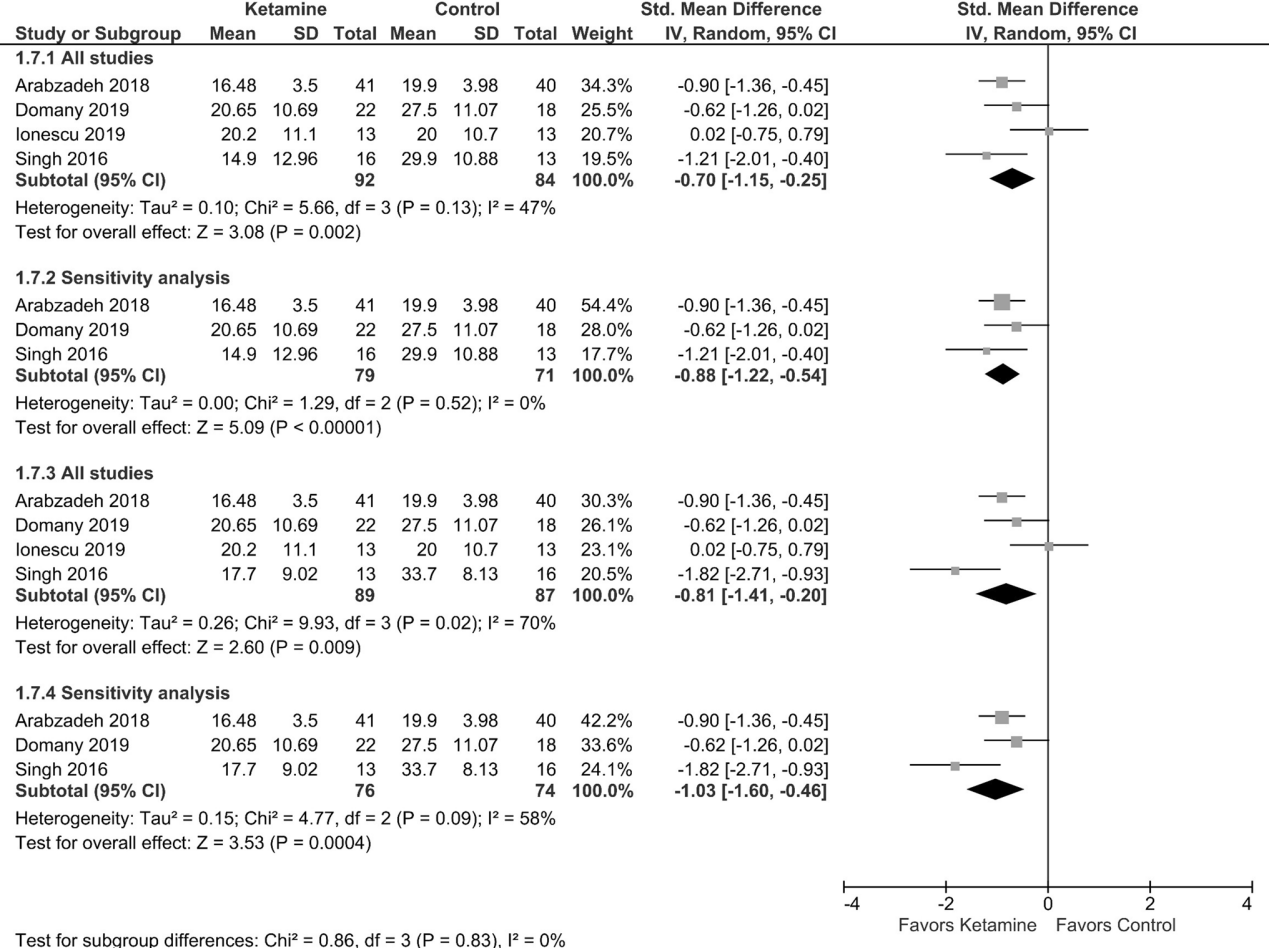
Effects of serial ketamine on depression rating scale at 2–3 weeks; data regarding twice-weekly (**a**) or thrice-weekly (**b**) dosing from the study by Singh et al. [32]. Sensitivity analysis excluded data from the study by Ionescu et al. [30]

Pooled data showed a significant reduction of depression severity scores at 2–3 weeks of repeated ketamine administration in comparison with placebo (Fig. 7). However, as in the case of single doses, leave-one-out analyses showed that exclusion of the study by Ionescu et al. [30], involving patients with suicidal thoughts, resulted in a more pronounced reduction of depression severity scores at 2–3 weeks of repeated ketamine administration (vs. controls) (Fig. 7), comparable to the effect size observed 24 h after single administration. It should be mentioned that both Grunebaum et al. [29] and Ionescu et al. [30] study involved patients with suicidal ideation, that in the latter were chronic (≥ 3 months).

**Fig. 8:**
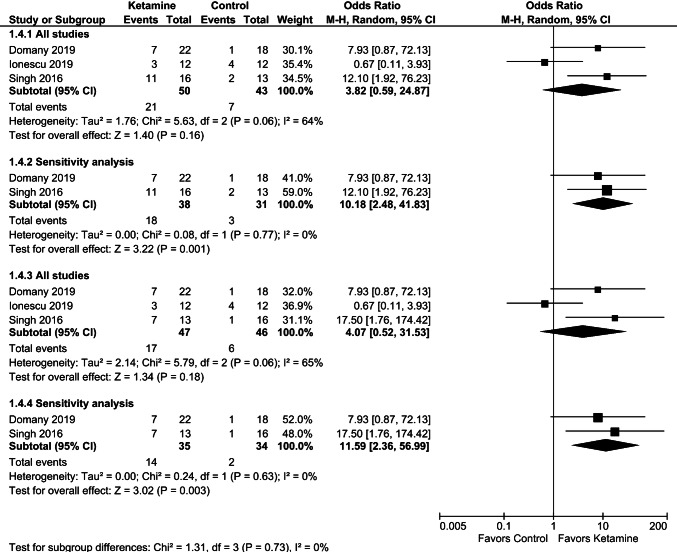
Effects of serial ketamine on response at 2–3 weeks, data regarding twice-weekly (**a**) or thrice-weekly (**b**) dosing from the study of Singh et al. [32]. Sensitivity analysis excluded data from the study by Ionescu et al. [30]

**Fig. 9:**
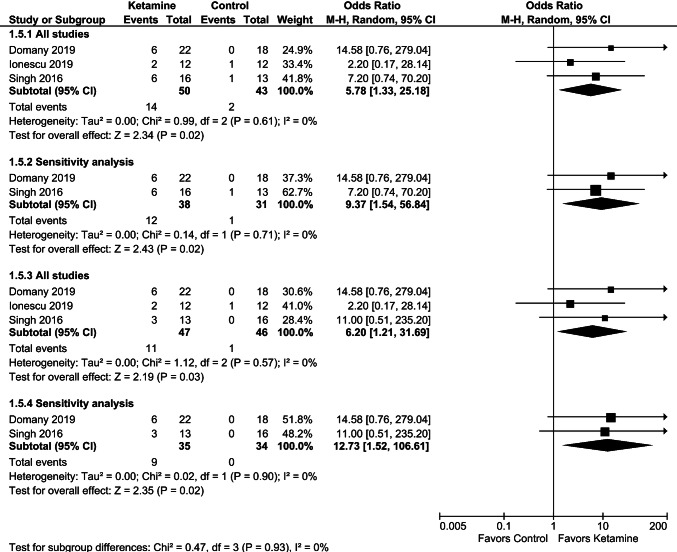
Effects of serial ketamine on remission at 2–3 weeks, data regarding twice-weekly (**a**) or thrice-weekly (**b**) dosing from the study of Singh et al. [32]. Sensitivity analysis excluded data from the study by Ionescu et al. [30]

As presented in Figs. 8 and 9, the pooled data of response and remission after excluding patients with suicidal thoughts [30] showed a significant difference in favor of ketamine over placebo at 2–3 weeks of repeated dosing.

## Discussion

Due to the very promising results of the original study [6], the antidepressant effects of ketamine have been extensively studied in the last years and some important and very recent RCTs in this field have been published between the years 2017 and 2019. The results of our meta-analysis are the most current and comprehensive pooled data of RCTs investigating single or repeated ketamine administration in adult patients with unipolar and bipolar depression. We attempted to answer the question whether antidepressant effects of single ketamine administration could be maintained during repeated ketamine treatment based on aggregated data from RCTs. Moreover, we aimed to fill the gap in the literature regarding prolonged ketamine exposure in patients with depression.

The meta-analysis of data for both continuous (depression severity scores) and dichotomous (response and remission rates) outcomes confirmed that significant efficacy advantage of single-dose ketamine vs. control was observed from 24 h to 7 days postadministration, with peak effects at 24 h and generally smaller-to-medium effects at 7 days. The results of our meta-analysis, which included also the most recent RCTs, are in line with previous meta-analyses [11, 17–19, 35, 36]. Significant benefits of single-dose ketamine vs. control were generally consistent among the subgroup of patients with TRD (approximately 50% of TRD patients reported response at 24 h after ketamine administration vs. 6% in control groups) when ketamine was used as monotherapy and as adjunctive to ongoing antidepressant therapy, as well as in unipolar and bipolar depression. However, most identified RCTs involved patients with unipolar depression, whereas only two studies were designed to assess the antidepressant effects of single-dose ketamine in patients with bipolar depression [8, 9] and none of the RCTs investigated repeated ketamine administration in bipolar disorder.

Primary studies as well as published meta-analyses showed that the effect of single-dose ketamine is short lived, and nearly all patients relapsed within 2 weeks postadministration [6–9, 11]. As patients with depression need long-term antidepressant effects, it is crucial to establish a strategy to maintain the antidepressant outcomes of ketamine for a longer time. Our results showed robust and significant advantages of serial ketamine administration over placebo at 2–3 weeks in terms of the reduction of depressive symptoms. Pooled data revealed a significant benefit of ketamine over placebo also in terms of response and remission, after exclusion of the study involving heavily pretreated patients with suicidal thoughts [30], due the reasons described below. As reported in included RCTs, antidepressant effects were maintained for about 1 week of follow-up after ketamine cessation [31, 32]. Although data from uncontrolled studies showed that most patients relapse after a few weeks (usually before the end of the third week) after cessation of even repeated ketamine treatment [12, 13, 15], a very recent RCT on intranasal esketamine showed a sustained improvement in the mean MADRS ratings over the 8-week follow-up after treatment cessation [16].

Although the antidepressant effects of ketamine have been known from many years, it is still unclear how ketamine elicits its effects in patients with depression. As ketamine is a potent noncompetitive glutamatergic *N*-methyl-d-aspartate (NMDA) receptor antagonists that binds to the phencyclidine binding site [37], its antidepressant effects are partially glutamate dependent. It has been proposed that through blockade of NMDA receptors on GABAergic interneurons ketamine reduce GABAergic transmission, which increases glutamate release and enhances the activation of α-amino-3-hydroxy-5-methyl-4-isoxazolepropionic acid (AMPA) receptors. These actions induce a signaling cascade that through brain-derived neurotrophic factor (BDNF) release and stimulation of mammalian target of rapamycin (mTOR) modifies the number and function of synaptic connections [38]. However, ketamine’s mechanism of action is more complex and includes also non-glutamatergic actions such as interactions with the monoaminergic system [39], potentiation of the inhibitory effects of GABA [40], and interactions with ion channels [41–43]. Ketamine has also antagonistic effect on cholinergic neurons [44]. Due to the complex mechanism of action, ketamine exerts multiple effects, including anesthetic, analgesic, antidepressant, and psychomimetic effects [45]. Different neuropharamacological actions are also involved in rapid and prolonged effects of ketamine exposure. While immediate effects are mostly connected with neuromodulation more delayed actions are connected with changes in gene expression and neuroplasticity. Given more complexity, different brain regions may be involved in the effects observed after single and repeated ketamine administration. Recent data have shown that single ketamine treatment of patients with TRD leads to increased regional cerebral blood flow (CBF) in the cingulate and primary and secondary visual areas [46]. On the other hand, repeated ketamine treatment leads to a decrease in regional CBF in the hippocampus and right insula [46], which was earlier reported on the basis of positron emission tomography (PET) findings [47].

Considering the complexity of neuropharamacological actions of ketamine, it is also interesting to discuss the selection of patients for inclusion in the analyzed RCTs, because patients with a high risk of suicide and concomitant psychiatric diagnosis were excluded from most trials. It cannot be ruled out that the antidepressant effects of ketamine differ in such specific subgroups of patients. Among the included RCTs, we found two studies (both on single-dose and repeated ketamine administration) in which the antidepressant effects of ketamine were very low [29, 30]. Both studies involved patients with suicidal ideation, and although ketamine has established antisuicidal effects, its effects on depression and suicidal thoughts are at least partially independent [29, 48]. As indicated by Grunebaum et al. [29], a reduction of suicidal ideation in patients with depression observed after a single ketamine infusion did not correspond with antidepressant effects. Other studies showed that adjunctive ketamine led to a reduction of suicidal ideation even in those patients whose depression did not remit [49, 50]. Of note, the results of another RCT involving patients with suicidal ideation also showed moderate antidepressant effects compared with control after a single-dose ketamine administration [51]. The results of an open-label study suggested that also the elevated level of treatment resistance is a potential factor explaining the lack of response to ketamine [52]. In line with this, a study by Ionescu et al. [30], in which ketamine did not outperform placebo in terms of antidepressant (and, in contrast to earlier trials, also antisuicidal) efficacy, involved heavily pretreated patients (more than six failed medication trials and more than 40% of patients failed ECT). Similar discrepancies in ketamine efficacy were also reported in uncontrolled studies. While up to six ketamine infusions led to response in 58%–68% of patients and remission in 42%–51% of patients [49, 53], the results of another study showed response and remission rates of 35.7% and 14.3%, respectively, despite an increase in ketamine dose to 0.75 mg/kg if the patient did not experience an improvement after the first 3 infusions of the 0.5 mg/kg dose [52]. As patients in the latter study were highly pretreated (the number of failed antidepressant trials ranged from 3 to 19), with 43% of patients failing previous ECT, this might have contributed to lower ketamine efficacy, as in the RCT by Ionescu et al. [30]. The discussed findings suggest that patients with suicidal ideation or those severely pretreated and resistant to multiple treatments may benefit less from ketamine treatment in terms of antidepressant effects or need higher doses to achieve antidepressant effects [52]. Future RCTs should recruit more heterogeneous populations to assess the impact of ketamine treatment on patient subgroups depending on baseline demographic data, comorbidities, and also use of various psychotropic medications. Of note, a recent uncontrolled clinical study confirmed high antidepressant efficacy of single ketamine infusion in patients with TRD who showed significant psychiatric comorbidity, with the 24-h response and remission rates of 54% and 42%, respectively [54].

Although another RCT included in the meta-analysis did not show antidepressant effects of ketamine vs. control [27], there is no clear explanation for these results. First of all, a significant placebo response was noted at all time points, and, in contrast to other trials, Downey et al. [27] reported less dissociative effects of ketamine, which were correlated with its antidepressant response [55]. The trial was conducted in untreated ambulatory participants with mild-moderate depression, and it was the only study where ketamine was infused for 60 min. In all other included RCTs where IV ketamine was used, infusions lasted over 30–40 min. However, the sessions of IV ketamine infusion as long as 100 min have been demonstrated to be effective in uncontrolled studies [14, 53].

Two of the RCTs investigating repeated ketamine administration used oral ketamine, and both of them confirmed that this route of administration was highly effective in amelioration of depressive symptoms at 2–3 weeks [31, 33]. Oral dosing of ketamine is a recognized route of administration in chronic pain management [56] and would be a more practical and acceptable delivery method than IV administration, especially for long-term use in patients with depression. Although the antidepressant effects of ketamine have been most extensively studied with the IV route of administration, some previous reports support oral ketamine use in depressed patients. The first positive report on the antidepressant effects of oral ketamine comes from hospice patients [57–59]. Oral ketamine was also effective in improving depressive symptoms in patients with mild-moderate depression and chronic pain in an RCT by Jafarinia et al. [60]. A recent retrospective series of TRD patients showed modest antidepressant efficacy of repeated oral ketamine; however, a relatively low starting dose of 50 mg every 3 days was used in this study [61]. Although the optimal dosing regimen and route of ketamine administration remain to be explored, the benefits are probably dose dependent in the range of 0.1–0.75 mg/kg when given IV [62]. The bioavailability of oral ketamine is around 24% [63], but given the potential cumulative effects of repeated dosing and concerns about safety of prolonged administration, oral ketamine at a dose of 1 mg/kg thrice weekly seems to be effective and safe [31] and is now under investigation in an RCT including patients with MDD [64].

## Limitations

This meta-analysis has several limitations, mostly discussed before. First of all, most studies in this review included small sample sizes. Heterogeneity was found with regard to the severity of depression in recruited patients, use of concomitant medications (continuing or tapering antidepressant therapy), documented resistance to antidepressant drugs, as well as depression scale used to assess antidepressant effects. Co-prescribed drugs might have an influence on ketamine effects; however, no details on ongoing antidepressant treatment were provided in the identified studies. Initiation of new antidepressant therapy with a drug of the same class both in the active and control arm of an RCT could reduce the effect of background antidepressant therapy, and this strategy was used in phase III clinical trials of intranasal esketamine [65, 66].

Not all included studies reported data at all the time points prespecified in this review, and data regarding the first period of crossover from two trials were not obtained. Some studies had missing data that were obtained eventually from graphs or clinical trials registry. Performed subanalysis raised also the question of effective masking in RCTs on ketamine. The use of placebo as a control is a limitation because the integrity of the blinding may be compromised. Ketamine administration causes transitory perceptual and dissociative disturbances that could lead to functional unblinding in patients and raters. Already the first published study showing antidepressant effects of ketamine reported that patients were readily able to distinguish between ketamine and placebo, owing to ketamine’s psychoactive effects [6]. The results of our meta-analysis showed a larger reduction (vs. controls) in depressive symptoms after single ketamine administration in placebo-controlled than midazolam-controlled trials. However, even when the active comparator (midazolam) was used in a primary study, 77% of patients were able to guess the assignment to IV ketamine at a dose of 0.5 mg/kg and around 37% were able to guess the assignment to midazolam [67]. As a result, some degree of functional unblinding may bias study outcomes in RCTs on ketamine, especially when placebo is used as control. Despite limitations, a large number of included studies as well as robust and generally consistent effects strengthen the results of the meta-analysis regarding single-dose ketamine. The main limitation regarding repeated ketamine administration is a relatively small number of studies with eligible data and their heterogeneity. As discussed above, those trials differ in terms of the route of ketamine administration and dosing regimen as well as the population included (highly pretreated patients with chronic suicidal ideation or patients with moderate to severe depression), which confused the results due to a possible difference in ketamine antidepressant effects in selected subgroups of patients. In one RCT where ketamine was used for acceleration of response to a newly initiated antidepressant, only data after 2 weeks of dosing could be used for the meta-analysis, as a recent meta-analysis showed that the onset of significant antidepressant effects of sertraline vs. placebo is observed in the third week at the earliest [34].

## Conclusions

The rapid and robust antidepressant effects of single-dose ketamine in patients with TRD suggest that ketamine is a promising candidate for an effective therapy in patients who do not respond to conventional treatment. Our findings showed that a single administration of ketamine reduces depressive symptoms and that the initial antidepressant effects of the drug are sustained during serial administration, with a significant efficacy advantage over placebo at 2–3 weeks. The most recent RCTs have shown that serial administration of oral ketamine has significant antidepressant effects in major depression. However, further studies are needed to assess long-term antidepressant effects of ketamine and to establish the optimal dose and route of administration. Our meta-analysis also highlighted a need for RCTs that would establish the antidepressant effects of ketamine in a selected group of patients, because highly pretreated individuals and those with suicidal ideation and resistance to multiple treatment approaches may benefit less from the antidepressant effects of ketamine than the general population.

## Electronic supplementary material

Below is the link to the electronic supplementary material.

Supplementary file1 (DOC 79 kb)
