# Supplementary material for: Efficacy of single and repeated administration of ketamine in unipolar and bipolar depression: a meta-analysis of randomized clinical trials
Source: Pharmacol Rep. 2020 Apr 16;72(3):543–62. doi: 10.1007/s43440-020-00097-z (PMC7329804; doi:10.1007/s43440-020-00097-z)
Supplement: Supplementary file 1 — Supplementary file1 (DOC 79 kb) [file 43440_2020_97_MOESM1_ESM.doc]

| **Section/topic** | **#** | **Checklist item** | **Reported on page #** |
| --- | --- | --- | --- |
| **TITLE** | | |  |
| Title | 1 | Identify the report as a systematic review, meta-analysis, or both. | Systematic review with meta-analysis |
| **ABSTRACT** | | |  |
| Structured summary | 2 | Provide a structured summary including, as applicable: background; objectives; data sources; study eligibility criteria, participants, and interventions; study appraisal and synthesis methods; results; limitations; conclusions and implications of key findings; systematic review registration number. | Abstract consists of: background; methods; results; conclusions; #2-3 |
| **INTRODUCTION** | | |  |
| Rationale | 3 | Describe the rationale for the review in the context of what is already known. | Unmet clinical needs for efficient drugs with a rapid onset of antidepressant effects.  A need for a meta-analysis that would elucidate the antidepressant effects of ketamine in selected group of patients; #4 |
| Objectives | 4 | Provide an explicit statement of questions being addressed with reference to participants, interventions, comparisons, outcomes, and study design (PICOS). | P - adult patients treated for major depression (unipolar or bipolar, treatment-resistant or not), with no restrictions on concomitant pharmacological or psychological treatments;  I - ketamine therapy at a fixed dose (single or repeated administration, with no restriction on the ketamine regimen used (e.g., dose or route);  C - placebo or non-antidepressant anesthetic;  O - change from baseline in depression severity scores on depression scales such as HDRS and/or MADRS (primary end point), also response and remission rates;  S – randomized controlled trial (crossover or parallel) assessing more than 5 patients; #5-6 |
| **METHODS** | | |  |
| Protocol and registration | 5 | Indicate if a review protocol exists, if and where it can be accessed (e.g., Web address), and, if available, provide registration information including registration number. | Protocol of this review has not been developed. |
| Eligibility criteria | 6 | Specify study characteristics (e.g., PICOS, length of follow-up) and report characteristics (e.g., years considered, language, publication status) used as criteria for eligibility, giving rationale. | Main inclusion criteria: PICOS as described above (no. 4); full-text, English-language articles; #5 |
| Information sources | 7 | Describe all information sources (e.g., databases with dates of coverage, contact with study authors to identify additional studies) in the search and date last searched. | Embase, Medline (via PubMed), Cochrane Central, Trip Database, clinical trials registries (www.clinicaltrials.gov, www.clinicaltrialsregister.eu) and hand-searching reference lists and possible contact with study author for missing data. Last search - February 22, 2019; #4 |
| Search | 8 | Present full electronic search strategy for at least one database, including any limits used, such that it could be repeated. | Keywords used in search strategy construction and methodological limits are presented in Table 1. (last updated: 22.02.2019) |
| Study selection | 9 | State the process for selecting studies (i.e., screening, eligibility, included in systematic review, and, if applicable, included in the meta-analysis). | The study selection was based on the titles and abstracts, and, finally, on full-text articles; #5; Figure 1. - PRISMA flow diagram for selection of studies:  -screening after duplicates removal;  -assessing for eligibility;  -included for qualitative analysis  - included for quantitative analysis (meta-analysis) |
| Data collection process | 10 | Describe method of data extraction from reports (e.g., piloted forms, independently, in duplicate) and any processes for obtaining and confirming data from investigators. | Data extracted by the first reviewer were verified by the second reviewer; #5;  For trials with a crossover design, results from the first period prior to the crossover were searched in published Cochrane meta-analyses or were requested from the authors of primary studies. Missing data were calculated from graphs in articles or searched in clinical trials registry; #6 |
| Data items | 11 | List and define all variables for which data were sought (e.g., PICOS, funding sources) and any assumptions and simplifications made. | Types of data sought: PICOS elements; details needed for risk of bias assessment. |
| Risk of bias in individual studies | 12 | Describe methods used for assessing risk of bias of individual studies (including specification of whether this was done at the study or outcome level), and how this information is to be used in any data synthesis. | Cochrane risk-of-bias tool was used at study level; see Figure 2. |
| Summary measures | 13 | State the principal summary measures (e.g., risk ratio, difference in means). | Effects measures: Standardized mean difference (SMD) and odds ratio (OR); #6 |
| Synthesis of results | 14 | Describe the methods of handling data and combining results of studies, if done, including measures of consistency (e.g., I^2^) for each meta-analysis. | Pooled SMD and OR with 95% confidence intervals were calculated for respectively, continuous and dichotomous outcomes; for both variables random effects model was applied; #6 |

Page 1 of 2

| **Section/topic** | **#** | **Checklist item** | **Reported on page #** |
| --- | --- | --- | --- |
| Risk of bias across studies | 15 | Specify any assessment of risk of bias that may affect the cumulative evidence (e.g., publication bias, selective reporting within studies). | Attempts of identification and explanation any potential biases are described in Chapter 4. and 4.1. |
| Additional analyses | 16 | Describe methods of additional analyses (e.g., sensitivity or subgroup analyses, meta-regression), if done, indicating which were pre-specified. | Sensitivity analysis and subgroup analyses were conducted;  - sensitivity analysis was scheduled for the primary outcome as leave-one-study-out and exclusion of crossover trials if data regarding the first period before the crossover were not obtained;  - subgroup analysis was planned for TRD, ketamine as monotherapy and as add-on to ongoing antidepressant therapy, placebo- and midazolam-controlled trials, unipolar and bipolar depression; #6-7 |
| **RESULTS** | | |  |
| Study selection | 17 | Give numbers of studies screened, assessed for eligibility, and included in the review, with reasons for exclusions at each stage, ideally with a flow diagram. | The flow diagram with reasons for studies’ exclusions is shown in Figure 1.  - 1418 records were screened (without duplicates);  - 61 were selected on the basis of titles and abstracts and assessed for eligibility;  - 20 studies (in 21 references) were included in the review and meta-analysis; #7 |
| Study characteristics | 18 | For each study, present characteristics for which data were extracted (e.g., study size, PICOS, follow-up period) and provide the citations. | The following data were extracted: study design; population, sample size; age of patients; details of intervention and regimen; concomitant therapy; definition of outcomes; baseline depression severity scores; results reported at specific time point.  Characteristic of each included study is presented in Table 2. |
| Risk of bias within studies | 19 | Present data on risk of bias of each study and, if available, any outcome level assessment (see item 12). | Review authors’ judgements about each risk of bias item for each included study are presented on Figure 2. (Risk of bias summary) |
| Results of individual studies | 20 | For all outcomes considered (benefits or harms), present, for each study: (a) simple summary data for each intervention group (b) effect estimates and confidence intervals, ideally with a forest plot. | Forest plots created in Review Manager tool for the outcomes are presented as Figures 3-9. |
| Synthesis of results | 21 | Present results of each meta-analysis done, including confidence intervals and measures of consistency. | Results of meta-analyses are presented in Chapter 3.2., 3.3., 3.4. and 3.6. |
| Risk of bias across studies | 22 | Present results of any assessment of risk of bias across studies (see Item 15). | Potential sources of bias are summarized in Figure 2. (Risk of bias summary) |
| Additional analysis | 23 | Give results of additional analyses, if done (e.g., sensitivity or subgroup analyses, meta-regression [see Item 16]). | Results of sensitivity and subgroup analysis are summarized in Table 3. (Effects of single-dose ketamine on depression rating scale at 24 hours, 3-4 days, and 7 days) |
| **DISCUSSION** | | |  |
| Summary of evidence | 24 | Summarize the main findings including the strength of evidence for each main outcome; consider their relevance to key groups (e.g., healthcare providers, users, and policy makers). | Summary of the key results and their significance are presented in Chapter 4. #11-15 and Chapter 5. #17 |
| Limitations | 25 | Discuss limitations at study and outcome level (e.g., risk of bias), and at review-level (e.g., incomplete retrieval of identified research, reporting bias). | Limitations of the review and quantitative synthesis are presented in Chapter 4.1.; #15-17 |
| Conclusions | 26 | Provide a general interpretation of the results in the context of other evidence, and implications for future research. | Conclusions are presented in Chapter 5. #17. Importance of the obtained results and their interpretation in the light of known literature are described in Chapter 4. |
| **FUNDING** | | |  |
| Funding | 27 | Describe sources of funding for the systematic review and other support (e.g., supply of data); role of funders for the systematic review. | The study was self- funding; #17-18 |

*From:*  Moher D, Liberati A, Tetzlaff J, Altman DG, The PRISMA Group (2009). Preferred Reporting Items for Systematic Reviews and Meta-Analyses: The PRISMA Statement. PLoS Med 6(7): e1000097. doi:10.1371/journal.pmed1000097

For more information, visit: **www.prisma-statement.org**.

Page 2 of 2
